# Rupture of Uterus

**Published:** 1869-10

**Authors:** 


### Rupture of Uterus.

By request, the following very interesting case is furnished by Dr. C. E. Parker, a practioner of thirty years experience, late from Beardstown, Ill., and now (having retired from the profession) a citizen of our own city:

Was called August 9th, 1862, at 2 o'clock P.M., to visit Mrs. R., residing about twelve miles from B. Mr. R. stated she had been in labor during the night, in charge of a neighboring midwife, who at noon reported "a portion of the child born, and something wrong." Arrived at 3½ P.M.; found the patient upon her back, in nearly a sitting position, chest flexed upon the abdomen; lower limbs drawn up. She reported her labor as having been very severe up to 11 o'clock, at which time, after a very violent expulsive effort, "something seemed to give way; all pain ceased, and a strange sickening sensation followed." The hand of the child protruded at 9 o'clock A.M. On manual examination, found a mass of material protruding, consisting of child's arm, a portion of placenta, and a large mass of something which I never met with before. On occular examination, found a large mass of the intestines protruding from the vulva; the partially expelled placenta was easily withdrawn; the hand cautiously introduced, the intestinal coils pushed up before it; found the child in the abdominal cavity; the left foot directly under the liver at its upper portion; the right on the opposite side of the abdomen; cautiously drawing them together, version was gradually produced; the arm slowly returning as the feet were drawn down. The body of the child was extracted readily, but the head brought down a large mass of the intestines, which were with difficulty returned and retained. On minute examination, found a rupture of the uterus on its left posterior lateral portion, the rupture extending through the os tincæ and a portion of the vagina. Allowed her to remain in the same position until all drainage had ceased, then placed her in a horizontal position, hips much elevated, and applied bandages and compresses, calculated to prevent further protrusions of the intestines, and ordered them to be kept saturated with cold water. Gave an opiate, and left her, supposing the injury in itself sufficient to produce death—my manipulations making that result only more certain. The next day Mr. R. called upon me to visit her again. My engagements, the distance, and extreme heat, prevented. Gave him a note to Dr. P., a very worthy and scientific practitioner, residing only three miles from the patient, requesting him to visit her and do all in his power to make her comfortable. On the 12th, received a note from Dr P., desiring me to meet him at Mr. R's. Found her quite comfortable, and desirous of sitting up. The question for decision, the admissibility of a cathartic. Examined, could detect the fissure in the vagina and os tincæ, but the union seemed firm, but little abdominal soreness, and no pain, except when turning in bed, which the temperature, 98° above, rendered frequently necessary. The carthartic operated kindly, and she was up and about as early as in her six previous labors. Sometime during 1865, she was safely delivered of a child without assistance, save what was rendered by neighboring women. This fact is vouched for by a lady who knew them intimately, and had seen the child repeatedly. I learned this but a short time since, they having removed to a neighboring county some distance, and I having left B. and the profession. In its first phase I consider the case unique; in its second, I have never heard its parallel.—*From Report on Obstetrics in Transactions of Illinois State Med. Society.*

